# The menace and outcome of severe acute malnutrition and diphtheria cases among paediatric under-five in-patients at Nguru North – east Nigeria

**DOI:** 10.1186/s12879-026-13606-x

**Published:** 2026-05-18

**Authors:** Surajudeen Oyeleke Bello, Tajudeen Lanre Ibrahim, Bello Abdullahi Ibrahim, Aisha Omotola Bello, Ikrama Ibrahim Hassan, Taofik Oluwaseun Ogunkunle

**Affiliations:** 1 Department of Paediatrics, Federal University of Lafia / Federal University Teaching Hospital, Lafia, Nasarawa State Nigeria; 2 Department of Paediatrics, Federal University of Health Sciences / Federal University of Health Sciences Teaching Hospital, Azare, Bauchi State Nigeria; 3 Department of Public Health, Maryam Abacha American University, Maradi, Niger; 4 Department of Community Medicine, Federal University of Lafia / Federal University Teaching Hospital, Lafia, Nasarawa State Nigeria

**Keywords:** Burden, In-patients, Outcome, SAM, Vaccine preventable disease

## Abstract

**Introduction:**

Severe acute malnutrition (SAM) is a disease of global public health concern, worse in developing countries, contributing to illnesses and deaths among under-five children. Diphtheria is a deadly disease with its burden alongside SAM, poorly known. This gap could impact negatively on policy formulation and interventions. In-hospital burden of under-five SAM and diphtheria with their outcome and associated factors were assessed in North-east Nigeria.

**Methods:**

A retrospective hospital-based cross-sectional study of children admitted for various illnesses at the Paediatric Emergency Unit, Paediatric Medical Ward and the Paediatric Infectious Diseases Unit of the Federal Medical Centre Nguru from 01/01/24–30/09/24. Data was analyzed using SPSS version 23. Univariate and multi-variates analyses were presented in tables and graphs, significant p was < 0.05.

**Results:**

There were 194 (12.7%) deaths from the total 1,530 under five admissions. Males accounted for 123 (63.4%) of these deaths. SAM contributed 854 (55.8%) to the total morbidities, while acute diarrhea disease and malaria were the other lead contributors to under-five morbidities with 207 (13.5%) and 130 (8.5%) cases respectively. SAM was the highest 139 (71.6%) contributor to mortalities, followed by Diphtheria 16 (8.2%), Acute Diarrhea diseases (ADDx) 13 (6.7%) and malaria 11 (5.7%). Diphtheria and SAM have the highest case fatality rates of 22.2% and 17.0% respectively. Age, admission duration and diagnosis were major determinants of outcome of care with aOR and CI of 4.24 (2.094–5.125), 6.92 (3.54–8.10) and 2.24 (1.18–3.67) respectively.

**Conclusions:**

We found high mortality rate, more males and children with SAM in this study. Most of the SAM patients were infants. SAM, ADDx and Malaria were the reasons for most admissions while SAM, Diphtheria, ADDx and Malaria were the major causes of deaths. Age, duration on admission and diagnosis were the main determinants of outcome of care. Underscoring the needs for early infant feeding and optimal complimentary feeding practices.

**Clinical trial number:**

Not applicable.

## Introduction

Malnutrition is defined as a “pathological condition arising from either an inadequate or excess food intake”, while the intake of nutritionally imbalanced diet impact negatively on the body [1]. High unemployment rate, poverty and illiteracy are some of the indirect factors associated with malnutrition among the vulnerable population like children [2, 3]. Severe acute malnutrition (SAM) is a major preventable disease of global public health concerns arising basically from denial of under-five children’s right [4, 5]. The enormity of this severe form of malnutrition is worse in the developing countries than the rest of the world [5, 6]. 

Despite modest decline in childhood mortalities (infant and under-five mortalities stood at 63 per thousand and 110 per thousand live births respectively as at 2024) occasioned by the efforts of governments and partners, the healthcare indices remained abysmally poor and unacceptably high [7]. While the international community is striving to attain the global nutrition target, most sub-Saharan African countries like Nigeria are still very far from its actualization [7, 8]. 

Malnutrition during the first 1000 days (which spans from duration of pregnancy up to the first two years) of life has far reaching implication on the child’s gross and fine motor development as well as overall longstanding intellectual and reproductive potentials [9]. Thus, ensuring optimal nutrition for lactating mothers is as important as that of the newborn [10]. Infants are to be exclusively breastfed for six months, followed by complementary feeding from six months, while breastfeeding is continued for twenty four months or more [11]. The maternal readiness for this optimal nutrition and immunization of children will be difficult, owing to the reliance of both on mothers and interconnectivities in an environment ravaged with banditry and insurgency with the attendant loss of life of breadwinners with displacements of vulnerable women and children [12, 13, 15, 18]. 

Diphtheria is one of the vaccine preventable diseases (VPD) caused by the Gram-positive bacillus *Corynebacterium diphtheriae* characterized by pharyngo-tonsillitis with an adherent pseudo-membrane of the pharynx [14, 15]. Approximately, one quarter of the cases develop myocarditis, and may also affect the peripheral nervous system often leading to death of the patients [15, 16]. This organism produces an extracellular toxin that is responsible for the havoc it causes, more-so in individuals with poor antibody levels to the toxins due to lack of or incomplete immunization [16]. Treatment of diphtheria consists of using the Diphtheria specific antitoxin (DAT) and the penicillin or erythromycin [15, 17]. 

Other causes of under – five deaths (diarrhea, pneumonia and malaria) do exist but with malnutrition as a common denominator [19, 20]. The burden of malnutrition and diphtheria in this vulnerable age groups is not well known in this locality. This knowledge gap could impact negatively on policy formulation and necessary interventions that such area may require to halt the trend. Hence, this study aimed at assessing the in-hospital burden of under-five severe acute malnutrition and diphtheria, a vaccine preventable disease as well as evaluate the outcome of care with the contributing factors in a tertiary health facility in North-east Nigeria.

## Materials and methods

Children admitted for various illnesses from the 1st day of January to the 30th of September 2024 were included in this study.

The Paediatric Medical Ward (PMW) of the Federal Medical Centre (FMC) Nguru, is the dedicated unit for stabilization and management of children with SAM. The Emergency Paediatrics Unit (EPU) is a two wings unit where one end was used for admitting the emergency cases and the other end for continued recuperation till discharge since the PMW is now being used for another case entirely. There is Paediatric six-bedded infectious diseases isolation unit (PIDU) where the Diphtheria cases were transferred to and managed within the hospital.

This was a retrospective hospital-based cross-sectional study. All eligible admissions during the study period were included (census of admissions).

All children admitted at either the EPU, PMW or the Paediatric infectious diseases isolation unit (for the clinically suspected diphtheria cases) of the centre were included in this study without exclusion. The doctor’s purposely kept ward register of all patients admitted were cross-checked with that of the Nurses and the Nutritionists (where applicable) to aid data completeness at the Emergency Paediatric Unit (EPU), Paediatric Medical Ward (PMW) and the paediatric infectious diseases isolation unit was used to get data of patients admitted from the first day of January to the 30th day of September 2024. Their ages in months, genders, presenting complaints, diagnosis, durations of hospital admission and outcome of care was retrieved by the researchers, assisted by a research assistant (a Nutritionist).

A total of 1,906 children were admitted within the study period, all children older than five years admitted at the EPU and babies managed at the Special Care Baby Unit SCBU (64 + 312 = 376) were excluded from this study. The well-kept doctors, nurses and nutritionist register used jointly and the short duration ensured data completeness at the PMW, EPU and PIDU respectively.

Anthropometric measurements were collected from case files. Nutritional status was graded in accordance with the WHO child growth standards. That is:

Severe Acute Malnutrition (SAM) = Weight – for – Lenght / Height < − 3 SD (Standard Deviation). While, Oedematous SAM = Weight – for – Lenght / Height < − 3 SD (Standard Deviation) + Oedema. There was no missing anthropometric data as all patients presenting at this unit (PMW) must have their anthropometric parameters done or even repeated if already done from the referring unit (e.g. EPU) as a prerequisite for justifying their diagnosis and admission to the ward.

The admitted SAM patients are co-managed by the doctors, nutritionists and the nurses with a care-giver per child, they are commenced on F-75 giving via a nasogastric tube for the first 48–72 h before changing to F-100 alongside other treatments like Oral Rehydration Solution when there is diarrheal diseases, antibiotics when there are features of sepsis, correction of electrolytes like potassium, correction of hypoglycemia and anaemia when detected as they are often looked out for. Others are prevention of hypothermia while monitoring vital signs, input / output charts and checking for return of appetite. Readilly Utilized Therapeutic Food (RUTF) are commenced once appetite returns and haematinics once there is no evidence of sepsis or it has resolved.

### Vaccine preventable diseases

Vaccine Preventable Diseases (VPD) referred to diseases that are potentially preventable when certain vaccines are given. Such diseases includes; Diphtheria, Tetanus, Poliomyelitis, Pertussis, Measles, Acute Baterial Meningitis and Tuberculosis [14]. 

### Standard case definitions [15, 21]

- **A Laboratory-confirmed case** of diphtheria is defined as a person who has a culture positive toxigenic strain of *Corynebacterium spp*. isolated by culture and is positive for toxin regardless of symptoms.
- **Clinical case**: This refers to a case of diphtheria that meets the criteria for a suspected case but does not have a confirmatory laboratory test result or a known epidemiological link to a laboratory confirmed.

Diphtheria diagnosis was clinical in this case with (Fever, Neck fullness (Bull-neck), Difficulty with breathing, Difficulty with swallowing and greyish – white membrane patch on the throat [20]. Some may be toxic looking and restless with hyper-extension of the neck or maintaining tripod position for support with or without drooling of saliva) as the hospital lacks the capacity for culture, polymerase chain reaction and toxin detection at the time.

All cases were managed using the penicillin injections (amoxicillin-clavulanic acid or crystalline penicillin) and with Diphtheria anti-toxin (DAT) which was available due to ongoing epidemics from a neighbouring states [15]. Children with pharyngitis may present with fever, reduced intake, cough, with or without vomiting and may require rehydration and electrolyte corrections. Thirteen of the SAM patient either had measles at admission or recovering from post measles with debility.

Sepsis in this study refers to children who presented with temperature instability (either high grade fever T > 38.5 °C or hypothermia T ≤ 35.0 °C), poor intake, excessive crying, vomiting, and or stooling. Thirteen of the SAM patient either had measles at admission or recovering from post measles with debility and some had tuberculosis.

Ethical approval was obtained from the Hospital Research Ethics Committee of the Federal Medical Centre, Nguru, Yobe State Nigeria FMC//CL.SERV/355/VOL.VI/7/10/2024 on the 15th of November 2024. Strict confidentiality and privacy of patient data was adhered to. The data was coded and entered into an excel spreadsheet before transferring to a Statistical Package for Social Sciences (SPSS) version 23. Result was presented in tables or graphs. Categorical variables was presented using frequencies and percentages. Multivariate regression analyses of variables (like age, gender and diagnosis) on the outcome of care were assessed. The A significant p value was set at *p* < 0.05. Deaths, discharges and others (like SAMA) among the in-patients were the outcome of care.

## Results

### Admissions and mortalities based on gender

Of the total 1,530 under five admissions within the nine months study period, there were one hundred and ninety four (12.7%) deaths. The mean age of this study population is 20.44 ± 10.97 months. Most of the admissions and the deaths were in children aged 1 – < 5 years.

Males accounted for 868 with a male to female ratio of 1.3:1. Admissions and deaths increased by quarter but with no significant association between gender and admissions per quarter (χ² = 3.21, *p* = 0.201). Deaths recorded among males was 123 (63.4%) of the total mortalities. The mortalities figure was highest in the months of July to September, but was not statistically significant (χ² = 2.36, *p* = 0.310) Table 1 below.

**Table 1:** Under five admissions and mortalities based on gender

Quarterly trends	Admissions by Gender					Deaths by Gender				
	Male *n* (%)	Female *n* (%)	Total *n* (%)	χ²	*p* value	Male *n* (%)	Female *n* (%)	Total *n* (%)	χ²	*p* value
Jan – March	223 (60.3)	147 ((39.7)	370 (24.2)	3.21	0.201	27 (64.3)	15 (35.7)	42 (21.6)	2.36	0.310
April – June	256 (57.3)	191 (42.7)	447 (29.2)			37 (57.8)	27 (42.2)	64 (33.0)		
July – Sept	389 (54.6)	324 (45.4)	713 (46.6)			59 (67.0)	29 (33.0)	88 (45.4)		
Total	868 (56.7)	662 (43.3)	1530 (100.0)			123 (63.4)	71 (36.6)	194 (100.0)		

**Fig. 1:**
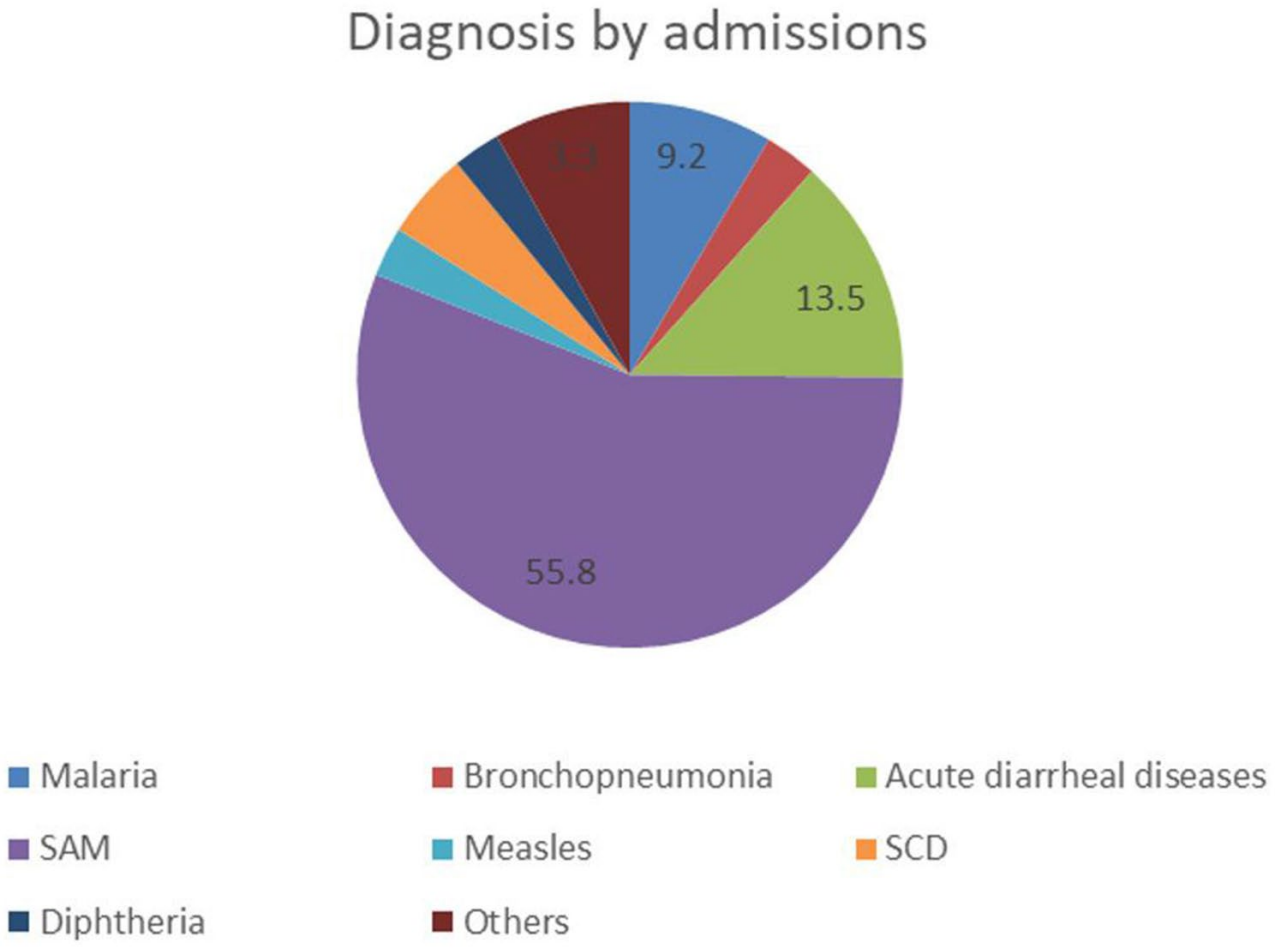
Distribution of admissions by diagnoses

Severe acute malnutrition SAM were 854 (55.8%) of the total under five admissions, while acute diarrhea disease was 207 (13.5%) and malaria, 130 (8.5%). While sickle cell diseases SCD 85 (5.6%), Bronchopneumonia 50 (3.3%), Measles 50 (3.3%) and Diphtheria 45 (3.0%). Others referred to (meningitis, bronchiolitis and tuberculosis etc) Fig. 1 above.

### Distribution of deaths by diagnoses

Diphtheria had the highest case fatalities 10 of 45 (22.2%), followed by SAM 145 of 854 (17.0%), Malaria 8 of 130 (6.2%), Acute Diarrhea Diseases 9 of 207 (4.3%) and Bronchopneumonia 2 of 50 (4.0%) Table 2 Below.

**Table 2:** Distribution of deaths by diagnoses

Variables	Admissions *n*	Deaths *n*	Case fatality rates (CFR %)
Severe Acute Malnutrition	854	145	17.0%
Diphtheria	45	10	22.2%
Malaria	130	8	5.7%
Acute Diarrhea Diseases	207	9	4.3%
Bronchopneumonia	50	2	4.0%
Measles	50	1	2.0%

### Complications / co-morbidities of severe acute malnutrition

Sepsis is the commonest (171, 20%) co-morbidity of SAM in this study. Others are bronchopneumonia (7.4%) and hypokalemia (6.2%) as shown in Table 3 below.

**Table 3:** Complications & co-morbidities of severe acute malnutrition

Variables	Frequency	Percentages
**Complications**		
Sepsis	171	20.0
Hypokalemia	53	6.2
Hypoglycemia	2	0.2
Anaemia	13	1.5
**Co-morbidities**		
Post measles	13	1.5
Bronchopneumonia	63	7.4
TB	10	1.2
HIV	3	0.4
Dysentry	8	0.9
Diarrhea diseases (acute & persistent)	518	60.7

**Fig. 2:**
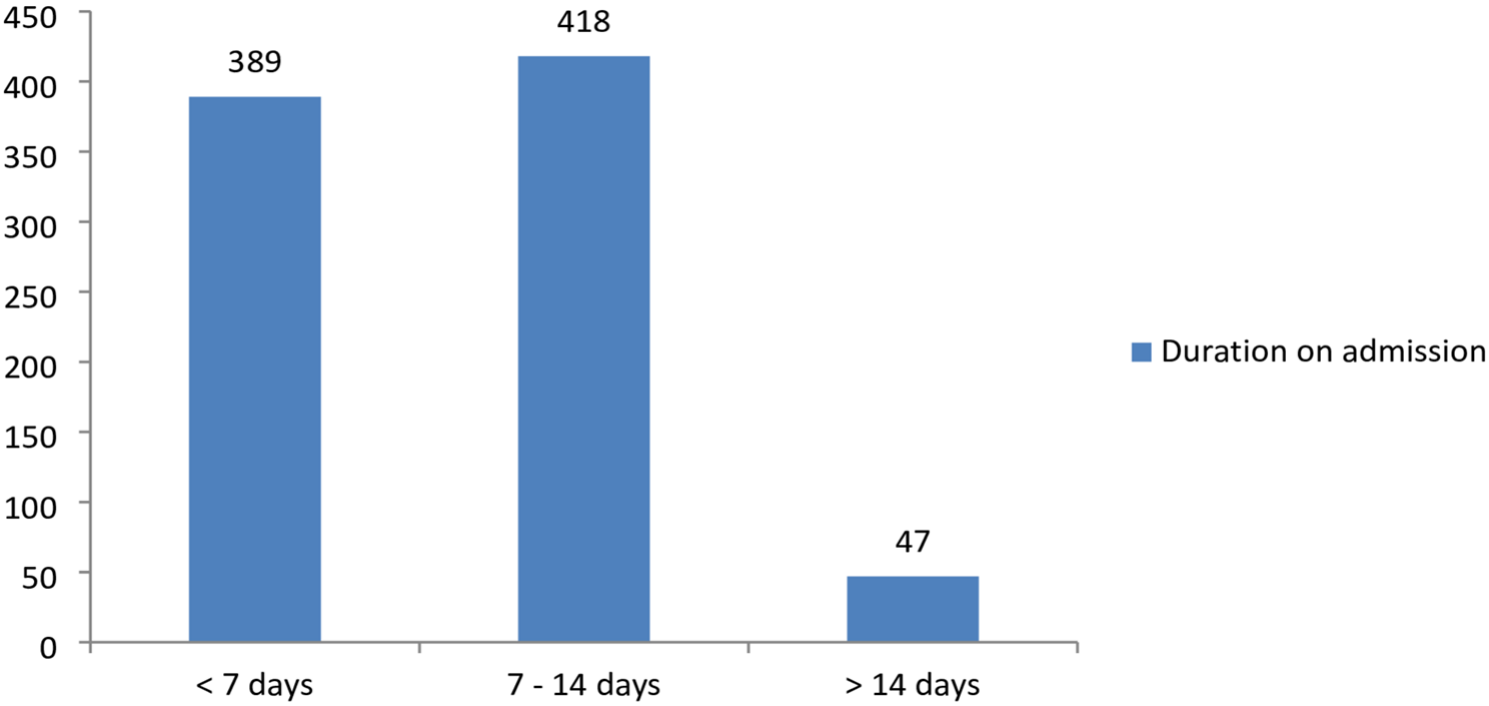
Duration of admission of sam clients

Most 418 (48.9%) of the SAM patients were on admission for between 7 and 14 days with only 47 (5.5%) staying beyond two weeks on admission. The median duration on admission is 11 days with inter-quartile range IQR of 5–17 days Fig. 2 above.

**Fig. 3:**
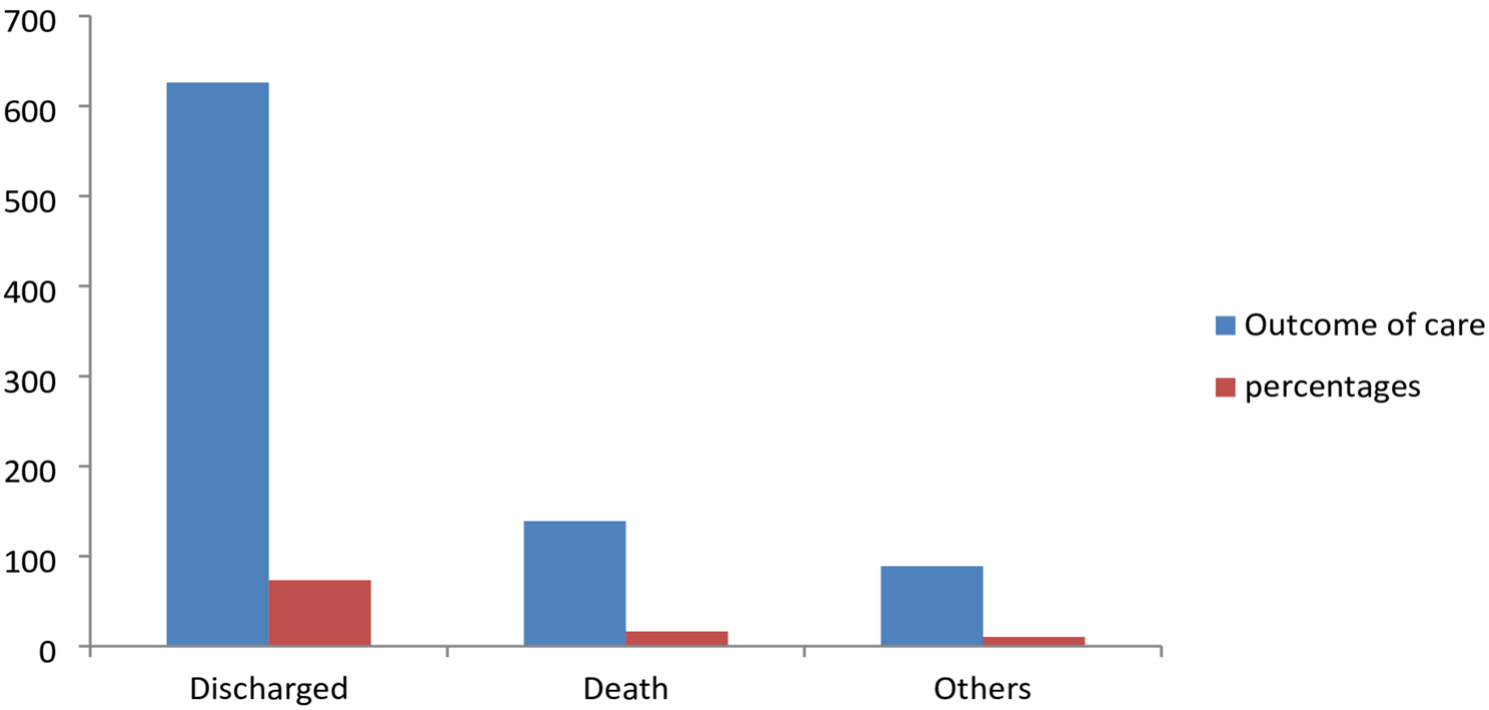
Outcome of care of the sam patients

Of the total 854 admitted SAM patients, 626 of 765 (81.8%) recovered and discharged home while 139 of 765 (18.2%) died on admission. Others (whose outcome were unknown to us) were ten (1.2%) DAMA and seventy nine who absconded (9.3%) Fig. 3 above.

### Factors determining the outcome of care

Age, duration on admission and diagnosis are statistically significant factors associated with the outcome of care in this study, with every increase in age of a child (in months), s/he is four times likely to survive the illness. Males are 1.2 times more likely to survive than the females, but not significant. Age and diagnosis at the time of admission are four time and twice associated with the outcome of care respectively (*p**** = 0.0001***) Table 4 below.

**Table 4:** Factors determining the outcome of care

Variables	χ²	B	cOR	aOR	*p* value	CI
Age (months)	255.230	1.444	4.581	4.236	0.0250	2.094–5.125
Gender (M/F)	1.515	0.196	1.223	1.216	0.2184	0.890–1.661
Diagnosis	245.336	0.805	2.512	2.236	**0.0023**	1.175–3.671

χ² = chi square, B = regression coefficient, cOR = Crude Odds Ratio, aOR = adjusted Odds Ratio, CI = Confidence Interval

## Discussion

Severe Acute Malnutrition (SAM) accounted for the highest morbidity and mortality in this study. Acute diarrheal diseases and malaria were also major contributors to morbidities, although their associated mortality was comparatively lower. Bronchopneumonia, measles, and diphtheria contributed relatively fewer admissions but still recorded deaths. Sickle Cell Disease (SCD) had notable admissions with minimal or no recorded mortality. There are more males in this study, and contributed to most of the mortalities. This is similar to earlier reports [22, 23]. Under five children accounted for most of the admissions, with most also found to be malnourished.

The relatively high mortality rate in this study can be attributed to the vulnerability of the study age group with a mean age of 20.44 ± 10.97 months and the high proportion of malnutrition. This under-five age group has the highest mortalities in most other studies in childhood like ours [22–25]. The mortality rate in the current study is higher than the report by Okeke et al. at a State Teaching Hospital in Orlu Imo State [22]. Similarly, it is higher than the finding from Niger Delta public health facility and much higher than that reported in Southwestern Nigeria [24, 25]. The difference could be attributed to the high admission rate in the current study despite a shorter study duration. Besides, the high rate of malnutrition in the present study in an environment with lower immunization coverage with some form of unrest [23]. The high fertility rates and overall poor healthcare seeking behavior in this part of the country could be contributory [26, 27]. Highest mortalities recorded in this study is similar to the Niger-Delta findings [23]. 

Severe acute malnutrition (SAM) accounted for most under-five admissions in this study. Acute diarrhea disease and malaria were the other common reasons for hospitalization in this study. Similarly, SAM, Diphtheria, Acute Diarrhea Diseases and malaria were the leading causes of mortalities in this study. SAM contributing to most of the deaths in this study is not surprising malnourished children are many times at risk of dying [28, 29]. 

Diphtheria had the highest case fatality in the present study, similar to reports from other studies [17, 22, 30]. Other earlier studies equally reported higher mortalities from diphtheria, malnutrition, malaria and acute diarrhea diseases [21, 24, 25]. The high cases of diphtheria accompanied by the high mortalities may be due to hard to reach areas and pre-existing poor healthcare seeking behavior of the people from this region [14, 15, 30]. The unacceptably high case fatality rate in the diphtheria cases is closely followed by SAM, who are mostly infants and immunocompromised hence unable to fight infections.

However, a sizeable number of the admitted SAM patients recovered and were discharged home, while few of the patients either absconded from the hospital or discharged themselves against medical advice. Most of the SAM patients stayed longer than a week on admission, similar to reports from earlier studies [30, 31]. Younger age, longer duration on admission and the diagnosis at admission are significant factors determining outcome of care in this study. Similar to other earlier studies [32, 33]. 

This study is limited for its retrospective nature that limited data to be collected and possibility of mis-classification of the “clinically diagnosed” diphtheria cases. Diagnostic challenge due to absence of confirmatory laboratory test as well as the restriction to nine months study period may be limitations as the panoramic view of diseases seasonality may be missed.

## Conclusions

1. Severe Acute Malnutrition (SAM) accounted for the highest morbidity in this study.
2. Acute diarrheal diseases and malaria were also major contributors to morbidity but with comparatively lower mortalities compared to diphtheria and SAM that together contributed almost half of the deaths.
3. Malaria, bronchopneumonia, measles, and diphtheria contributed relatively fewer admissions but recorded mortalities.
4. Age and diagnosis at admission are significant factors determining outcome of care.

### Recommendations

1. Intensifying community – based prevention with early detection and management of Severe Acute Malnutrition (SAM).
2. Improve public health interventions such as improved sanitation, access to clean water, hand hygiene, oral rehydration therapy, promoting exclusive breastfeeding, optimal complimentary feeding practices with locally available diets and use of insecticide-treated mosquito nets to curb both diarrhea diseases and malaria.
3. Enhancing prompt detection and treatment of diseases such as malaria, bronchopneumonia, and measles.
4. Strengthening routine immunization programs to eliminate vaccine-preventable diseases such as measles and diphtheria.
5. Continuous training and capacity building of healthcare workers in the management of pediatric emergencies.

## Data Availability

Data for this study can be extracted from the various unit’s well-kept register and it is also available upon request through the Hospital Medical Records / Health Information Management Staffs.

## Abbreviations

ADDx: Acute diarrheal diseases
BPN: Bronchopneumonia
DAT: Diphtheria anti-toxin
EPU: Emergency paediatric unit
FMC: Federal medical centre
HIV: Human immunodeficiency virus
MUAC: Mid upper arm circumference
NPI: National programme on immunization
PIDU: Paediatric infectious diseases unit
PMW: Paediatric medical ward
SAM: Severe acute malnutrition
SCD: Sickle cell disease
VPD: Vaccine preventable diseases
